# Collagen-targeted PET imaging for progressive experimental lung fibrosis quantification and monitoring of efficacy of anti-fibrotic therapies

**DOI:** 10.7150/thno.106367

**Published:** 2025-01-13

**Authors:** Alexandre Magno Maneschy Dias, Olivier Burgy, Mathieu Moreau, Victor Goncalves, Lenny Pommerolle, Romain Douhard, Alan Courteau, Alex Helbling, Mélanie Guillemin, John Simonet, Alexandra Oudot, Carmen Garrido, Philippe Bonniaud, Françoise Goirand, Bertrand Collin, Pierre-Simon Bellaye

**Affiliations:** 1 Centre Georges-François Leclerc, Service de Médecine Nucléaire, Equipe IMATHERA, 1 rue du professeur Marion, Dijon, France.; 2 INSERM U1231, Equipe HSP-pathies, Faculty of Medicine and Pharmacy, University of Bourgogne-Franche Comté, Dijon, France.; 3 Centre de Référence Constitutif des Maladies Pulmonaires Rares de l'Adultes de Dijon, réseau OrphaLung, Filère RespiFil, Centre Hospitalier Universitaire de Bourgogne, Dijon, France.; 4 Université de Bourgogne, UFR santé, 21000 Dijon France.; 5 Institut de Chimie Moléculaire de l'Université́ de Bourgogne, UMR CNRS 6302, Université de Bourgogne Franche-Comté, 21000 Dijon, France.; 6 Institut Universitaire du Poumon Dijon-Bourgogne, CHU Dijon, Dijon, France.; 7 Centre Georges-François Leclerc, Early Phase Unit, Dijon, France

## Abstract

Idiopathic pulmonary fibrosis (IPF) is a progressive disease characterized by an excessive collagen deposition ultimately leading to tissue stiffening and functional decline. Beyond IPF, other progressive pulmonary fibrosis are often associated with connective tissue diseases and may develop in ∼18-32% of patients. Therapeutic options are limited to nintedanib and pirfenidone which are only able to reduce fibrosis progression without curing it. The current lack of biomarker to accurately assess and predict disease progression and therapy efficacy for IPF remains a major clinical concern.

**Methods:** In our study, collagen deposition was monitored in bleomycin-induced lung fibrosis in mice by *in vivo* molecular imaging using a collagen-targeted radiopharmaceutical, [^68^Ga]Ga-NODAGA-collagelin. Fibrosis progression was also monitored using computed tomography, the gold standard technique to detect lung fibrosis in patients.

**Results:** We demonstrated that the bleomycin-induced increase in collagen lung content can be accurately quantified by [^68^Ga]Ga-NODAGA-collagelin PET imaging in correlation with disease stage and severity. The lung uptake of [^68^Ga]Ga-NODAGA-collagelin was mainly found in fibrotic areas of lungs in bleomycin-receiving mice. Most interestingly, [^68^Ga]Ga-NODAGA-collagelin PET imaging allowed the *in vivo* non-invasive monitoring of nintedanib efficacy as well as the anti-fibrotic effect of the JAK inhibitor, tofacitinib.

**Conclusion:** Thus, collagen-targeted PET imaging appears as a promising non-invasive tool for staging, monitoring and prediction of disease progression and therapy efficacy towards personalized medicine in IPF.

## Introduction

Idiopathic Pulmonary Fibrosis (IPF) is a rare and devastating progressive interstitial lung disease (ILD) with a poor median survival (5 years, 1) and an increasing prevalence 2, 3. Beyond IPF, other progressive fibrosing ILDs are often associated with connective tissues diseases, such as systemic sclerosis and rheumatoid arthritis (RA), and may develop in ∼18-32% of patients with ILDs. Currently, no curative treatment is available to reverse or even stop the disease progression. Only nintedanib and pirfenidone are commonly used and decelerate lung function decline 1, 4. IPF is characterized by the usual interstitial pneumonia (UIP) histopathological and imaging pattern and because of its rare and diffuse form, early diagnosis is a clinical challenge. Despite increasing knowledge, the physiopathology of IPF remains misunderstood and finding new therapeutic targets and associated biomarkers are major clinical concerns. While High-Resolution Computed Tomography (HRCT) considerably improved IPF diagnosis 2, a significant number of patients requires invasive procedures such as lung biopsy with a significant associated morbidity and mortality. As there is currently no tool to predict disease progression in IPF patients, the discovery of non-invasive biomarkers to promote early diagnosis and monitor fibrosis evolution is key to improve patients' outcome and therapy efficiency. The unpredictable interpersonal-variability of current anti-fibrotic drugs, pirfenidone and nintedanib, in term of efficacy is another evidence that IPF biomarkers are desperately needed. Accordingly, new imaging predictive biomarkers may constitute candidates to ameliorate patients' care towards personalized management of IPF patients.

*In vivo* molecular imaging may allow the non-invasive accessibility to early disease biomarkers thus improving early screening of patients who are at risk for developing progressive fibrosing interstitial lung disease, tracking disease progression and monitoring therapy effectiveness. The hallmark of pulmonary fibrosis is an abnormal and massive increase in extracellular matrix (ECM) deposition, mainly collagen, which disrupts the alveolar architecture and may reflect disease progression. As a result, these ECM components may be excellent indicators for early lung fibrosis development. Radiotracers that target specific ECM fibers, like collagen I/III, fibronectin and integrins, have undergone development and have demonstrated positive preclinical and clinical results in fibrotic disorders 5-9. Nevertheless, whether imaging of ECM fibers is a predictive marker of disease progression and efficacy of anti-fibrotic therapies remains unknown. Collagelin is a cyclic peptide with micromolar affinity to collagen I and III which binds to the triple helix structure of collagen 10. Collagelin was radiolabeled with technetium-99m for single photon emission tomography (SPECT) imaging and demonstrated its potential to specifically detect fibrosis through collagen binding in animal models 10. More recently, Velikyan *et al*. developed collagelin analogues with two chelating agents, 2-(4,7-bis(2-(tert-butoxy)-2-oxoethyl)-1,4,7-triazonan-1-yl)acetic acid (NO2A) or 4-(4,7-bis(2-(tert-butoxy)-2-oxoethyl)-1,4,7-triazacyclononan-1-yl)-5-(tert-butoxy)-5-oxopentanoic acid (NODAGA), allowing gallium-68 (^68^Ga) complexation for PET imaging 11, 12.

In this context, the current study evaluates the potential of [^68^Ga]Ga-NODAGA-collagelin *in vivo* PET imaging to monitor disease stage/progression in a preclinical model of pulmonary fibrosis in comparison with computed tomography (CT). In addition, the use of [^68^Ga]Ga-NODAGA-collagelin *in vivo* PET imaging to quantify the efficacy of novel and existing anti-fibrotic therapies was evaluated. Finally, the use of [^68^Ga]Ga-NODAGA-collagelin *in vivo* PET imaging as a predictive biomarker of fibrosis progression and therapy efficacy was also assessed.

## Material and Methods

### Animal experiments

All animal studies were conducted in accordance with the legislation on the use of laboratory animals (directive 2010/63/EU) and were approved by accredited Ethical committee (C2ea Grand Campus n°105) and the French Ministries of Research and Agriculture (project #30381). Eight-week-old C57BL/6 mice received at D0 a single intratracheal injection of 2 mg/kg of BLM (Santa Cruz biotechnology, USA) or NaCl (Controls) under anaesthesia (3% isoflurane). BLM-induced lung fibrosis is characterized by an initial inflammatory phase lasting from D0 to D7 which moves on to a fibrotic phase from D7 to D23. When indicated animals were treated with nintedanib (Ofev^TM^, 60mg/kg) or tofacitinib (Xeljanz^TM^, 30mg/kg) by daily gavage during the fibrotic phase of BLM-induced lung fibrosis from D8 to D22 as recommended 13.

### *In vivo* imaging

A pilot study was performed on NaCl- and BLM-receiving mice at D21 by dynamic PET/CT imaging with [^68^Ga]Ga-NODAGA-collagelin (NaCl, n = 1; BLM, n = 1). Mice were maintained under anesthesia (1.5% isoflurane) and placed on an imaging heated bed inside a BioPET^TM^/CT (Sedecal, Spain). Mice were then injected intravenously by the tail vein with [^68^Ga]Ga-NODAGA-collagelin (5MBq, 1.5 nmol/mouse) 2 minutes before start of imaging. A dynamic PET imaging (lung-centered, 250-700 keV) was performed from 2 minutes post-injection (p.i.) to 75 minutes p.i. A CT scan of a lung-centered region was then obtained (500 ms, 45 kV, 180 projections, pitch 1, binning 1:4).

In another experiment, NaCl- and BLM-receiving mice at several stage underwent dynamic PET/CT imaging with [^68^Ga]Ga-NODAGA-collagelin (NaCl, n = 15; BLM, J14, n = 3 and BLM, J21, n = 13, Suppl. Figure S3A) from 15 minutes p.i. to 75 minutes p.i. Additionally, a group of mice received a concomitant injection of [^68^Ga]Ga-NODAGA-collagelin (5MBq, 1.5nmol/mouse) with an excess (x100) of unlabelled collagelin (Blocking, n = 3). After the last imaging at D21, mice were sacrificed and lungs, blood, bladder, liver, spleen, heart, muscle and kidneys were harvested for *ex vivo* quantification of radioactivity using a γ-counter (Wizard, Perkin Elmer). Lungs were collected in 10% formalin for further histological analysis.

In other experiments, longitudinal imaging of lung fibrosis was performed on NaCl- (n = 4) and BLM-receiving mice treated with vehicle (n = 4), nintedanib (n = 4) or tofacitinib (n = 4) by successive PET/CT with [^68^Ga]Ga-NODAGA-collagelin before BLM installation, at D8, D15 and D22 following the same imaging protocol as described above (Figure S3B).

### Image analysis

All PET/CT fusion images were obtained using the VivoQuant^TM^ software (Invicro, USA). Each image was visually interpreted and 3D regions of interest (3DROI) corresponding to the lungs were manually drawn for CT quantification and to determine their radioactivity content. Dynamic PET images were reconstructed using a 2 minutes interval. Injected doses per animal were measured at the time of injection in MBq. Lung Radioactivity content was expressed in MBq, converted to percentage of injected dose per gram of tissue (%ID/g). All images were decay corrected for quantification. In addition, a semi-automatic segmentation of 3DROI was performed on CT scans as follows: normal lung density (-800 to -100 HU) corresponding to aerated lung areas and high lung density (-100 to 300 HU) corresponding to non-aerated/aerated lung areas as previously described 14. This semi-automatic segmentation allowed the independent quantification of the radioactivity content of [^68^Ga]Ga-NODAGA-collagelin (%ID/g) in normal and high density lung tissue respectively.

### Histology and collagen quantification

For histochemical assay, the amount of collagen in paraffin-embedded tissue sections was quantified by staining with PicroSirius Red as previously described 15. In addition, Masson trichrome staining were performed on D21 lung section from NaCl- and BLM-receiving mice treated or not with nintedanib or tofacitinib.

### Autoradiography

After deparaffination (Xylene) and antigen unmasking (30 min in citrate buffer pH 6), lung sections from mice receiving NaCl or BLM (D21) or human lung biopsies were saturated (BSA 8%) and incubated for 1h with [^68^Ga]Ga-NODAGA-collagelin (1 MBq, 1.5 nmol/slide). After 4 washes with cold DPBS, slides were exposed to phosphor imaging plates (Fuji imaging plates, Fujifilm). After 2-3 h of exposure time, the imaging plates were scanned and the autoradiograms were obtained with a phosphor imaging system (GE, Amersham, Molecular Dynamics) and the images were analysed for count densities. Data were used to calculate autoradiographic signal intensity in the lung sections.

### Statistical analysis

Comparison between two groups were performed using the Mann-Whitney non-parametric t tests. Comparison between multiple groups have been performed using the Kruskal-Wallis non-parametric ANOVA tests with a Bonferroni post-hoc test. A p < 0.05 was considered significant (*p < 0.05, **p < 0.01, ***p < 0.001). Results are presented as median ± interquartile range.

## Results

### Collagen-targeted PET imaging allows experimental lung fibrosis staging

NODAGA-collagelin was synthetized (Figure S1A) and radiolabeled with a radiochemical purity > 95% (Figure S1B). The stability of [^68^Ga]Ga-NODAGA-collagelin in plasma was 92.6 ± 0.41% at 1h and 88.3 ± 0.28% at 2h (Figure S1C).

Dynamic PET imaging was performed on NaCl- and BLM-receiving mice following IV injection of [^68^Ga]Ga-NODAGA-collagelin from 2 minutes up to 75 minutes p.i. in order to determine the optimal window of [^68^Ga]Ga-NODAGA-collagelin lung uptake to detect lung fibrosis. Lung uptake of [^68^Ga]Ga-NODAGA-collagelin rapidly increased at 2 minutes p.i. in both NaCl- and BLM-receiving mice (Figure S2A). A rapid decrease in [^68^Ga]Ga-NODAGA-collagelin lung uptake occurred between 2 minutes and 12 minutes p.i. (Figure S2A). From 12 minutes p.i. [^68^Ga]Ga-NODAGA-collagelin lung uptake in NaCl-receiving mice continue to decrease up to the baseline while it remained higher and slightly increasing in BLM-receiving mice (Figure S2A). From these results the window from 15 minutes to 75 minutes p.i. was used as optimal to quantify [^68^Ga]Ga-NODAGA-collagelin lung uptake.

**Figure 1:**
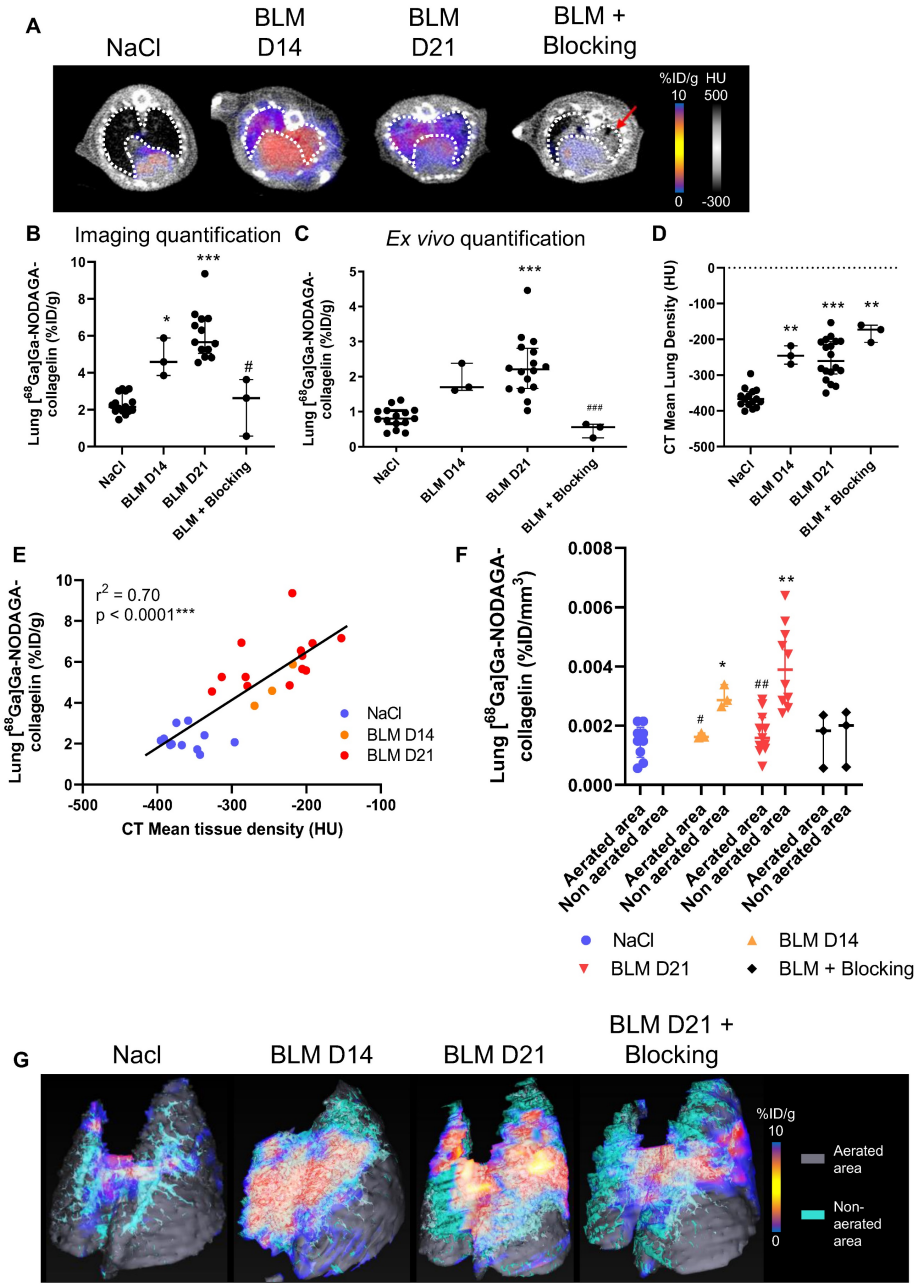
**[^68^Ga]Ga-NODAGA-collagelin is able to detect several stages of BLM-induced lung fibrosis.** A/ Representative lung PET/CT images with [^68^Ga]Ga-NODAGA-collagelin of NaCl- and BLM-receiving mice at D14 and D21. Red arrow highlights a fibrotic area. B/ Graph represents the [^68^Ga]Ga-NODAGA-collagelin lung uptake in %ID/g of NaCl- and BLM-receiving mice at D14 and D21 quantified on PET images. C/ Graph represents the [^68^Ga]Ga-NODAGA-collagelin lung uptake in %ID/g of NaCl- and BLM-receiving mice at D14 and D21 measured by gamma counting. Results are presented as median ± interquartile range, NaCl, n = 15; BLM, D14, n = 3; BLM, D21, n = 13. D/ Graph represents the mean lung density quantified on CT images of NaCl- and BLM-receiving mice at D14 and D21. Results are presented as median ± interquartile range, NaCl, n = 15; BLM, D14, n = 3; BLM, D21, n = 13. E/ Correlation between mean lung densities (HU) measured on CT images and [^68^Ga]Ga-NODAGA-collagelin lung uptake (%ID/g) of corresponding lungs measured on PET/CT. F/ Graph represents the [^68^Ga]Ga-NODAGA-collagelin lung uptake in % ID/g of NaCl- and BLM-receiving mice at D14 and D21 in aerated and non-aerated lung areas (segmented on CT images). G/ Representative 3D rendering of the localization of [^68^Ga]Ga-NODAGA-collagelin uptake in the aerated (gray) and non-aerated areas (blue) of the lungs from NaCl- and BLM- receiving mice at D14 and D21. A-F/ Results are presented as median ± interquartile range, NaCl, n = 15; BLM, D14, n = 3; BLM, D21, n = 13. Stars (*) are representative of comparison of each group with NaCl group and hashs (#) are representative of statistical comparison of blocking group with BLM D21 group. Differences between groups were compared using Kruskal-Wallis non-parametric ANOVA. *^(#)^p < 0.05, **^(##)^p < 0.01, ***(###)p < 0.001.

NaCl and BLM-receiving mice underwent PET/CT imaging with [^68^Ga]Ga-NODAGA-collagelin at several stages of experimental fibrosis (Figure S3A). The [^68^Ga]Ga-NODAGA-collagelin lung uptake was significantly increased in BLM-treated mice at D14 and D21 compared to controls (Figure 1A-B and S2B). *Ex vivo* radioactivity quantification of the lungs confirmed the increase in [^68^Ga]Ga-NODAGA-collagelin lung uptake at D14 and D21 in BLM-treated animals (Figure 1C). Similarly, lung CT of BLM-receiving mice showed an increase in fibrotic consolidations compared with control mice with an increase in mean lung density (MLD) at D14 and D21 (Figure 1D). The concomitant administration of an excess of unlabeled collagelin (blocking conditions) induced a significant decrease in [^68^Ga]Ga-NODAGA-collagelin lung uptake in fibrotic mice (Figure 1A-B-C). Interestingly, [^68^Ga]Ga-NODAGA-collagelin lung uptake on PET/CT significantly correlated with MLD measured on CT (Figure 1E). Similarly, *ex vivo* [^68^Ga]Ga-NODAGA-collagelin lung uptake correlated with collagen content measured by histology (picrosirius red quantification) in BLM-receiving mice at D21 (Figure S2C). In addition, gamma counting demonstrated an increase in lung-to-blood and lung-to-muscle ratio at D14 and D21 in BLM-receiving mice compared to controls (Figure S2D). Most interestingly, the increase in [^68^Ga]Ga-NODAGA-collagelin lung uptake was significantly increased in non-aerated areas of the lungs determined on CT at D14 and D21 compared with aerated areas which showed only minimal [^68^Ga]Ga-NODAGA-collagelin uptake (Figure 1F-G).

**Figure 2:**
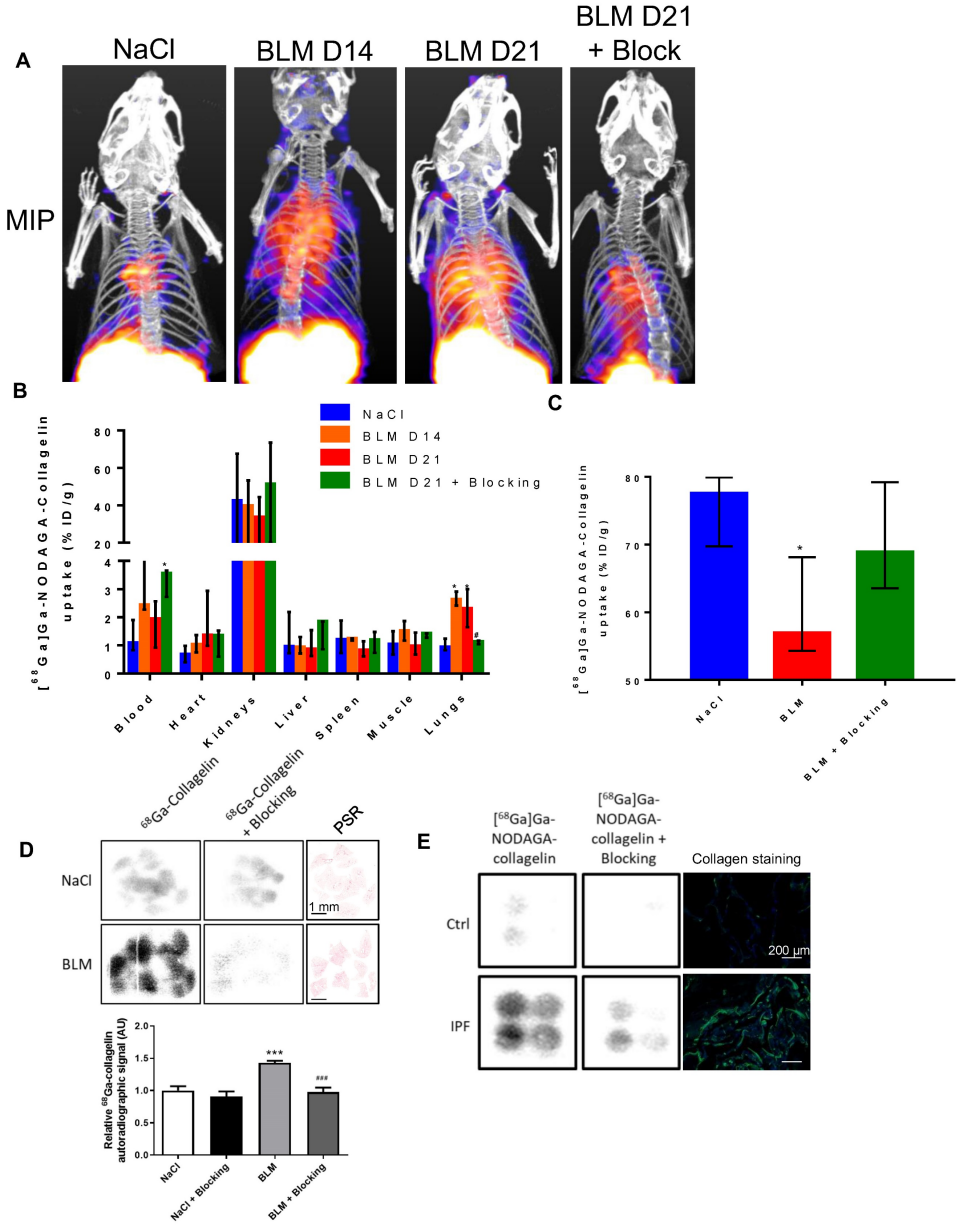
**[^68^Ga]Ga-NODAGA-collagelin biodistribution and autoradiography.** A/ Representative Maximal Intensity Projection of PET images of [^68^Ga]Ga-NODAGA-collagelin in NaCl- and BLM-receiving mice at D14 and D21. B/ Global biodistribution of [^68^Ga]Ga-NODAGA-collagelin in NaCl- and BLM-receiving mice at D14 and D21 (and blocking group). Results are presented as median ± interquartile range, NaCl, n = 12; BLM, D14, n = 3; BLM, D21, n = 12, blocking, n = 3. C/ Elimination of ^68^Ga]Ga-NODAGA-collagelin in NaCl- and BLM-receiving mice at D21 (and blocking group). Results are presented as median ± interquartile range, NaCl, n = 6, BLM, D21, n = 6, blocking, n = 3. B and C/ * indicates statistic comparison with NaCl group; #, statistical comparison between BLM D21 and BLM D21 + blocking groups. Kruskal-Wallis non-parametric ANOVA. *^(#)^p < 0.05. D/ ^68^Ga-collagelin autoradiography images, picosirius red (PSR) staining, and quantification on lung sections from mice receiving NaCl or BLM (D21). Results are presented as median ± interquartile range, n = 5 for all groups, scale bar = 1 mm. *, indicates statistical comparison of each group with the NaCl group; #, statistical comparison of the blocking group with the BLM group. Kruskal-Wallis non-parametric ANOVA. ***^(###)^p < 0.001. E/ ^68^Ga-collagelin autoradiography images and collagen immunofluorescence (Green = Collagen, blue = DAPI) on lung sections from IPF patients and controls, scale bar = 200 µm.

The global biodistribution of [^68^Ga]Ga-NODAGA-collagelin demonstrated an elimination route through the kidneys. In BLM-receiving animals, an increase in blood, heart and lungs was measured compared to NaCl animals (Figure 2A-B). The elimination rate of [^68^Ga]Ga-NODAGA-collagelin was decreased in BLM-receiving mice compared to controls (Figure 2C).

Similar results were obtained by autoradiography on lung sections from NaCl and BLM-receiving mice and patients with IPF (and controls) which showed a higher autoradiographic signal of [^68^Ga]Ga-NODAGA-collagelin in lung sections from BLM-receiving mice and IPF patients compared with controls (Figure 2D-E). Similarly, the concomitant addition of an excess of unlabeled collagelin (blocking conditions) strongly reduced [^68^Ga]Ga-NODAGA-collagelin autoradiographic signal demonstrating the specificity of the binding (Figure 2D-E).

### *In vivo* [^68^Ga]Ga-NODAGA-collagelin PET imaging is a useful tool to monitor nintedanib efficacy

**Figure 3:**
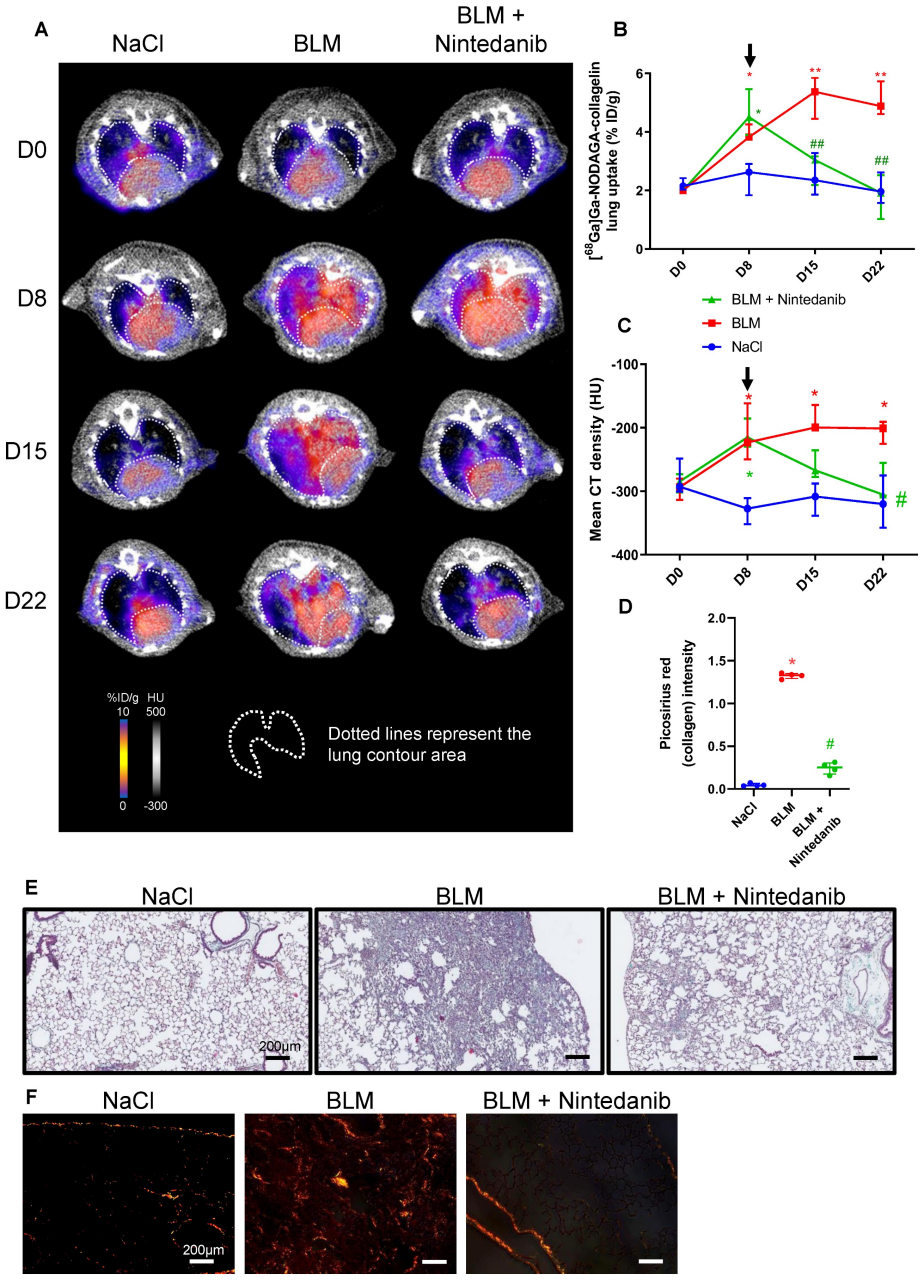
**Nintedanib reduces lung fibrosis and [^68^Ga]Ga-NODAGA-collagelin lung uptake.** A/ Representative [^68^Ga]Ga-NODAGA-collagelin PET/CT images of NaCl- and BLM-receiving mice treated or not with nintedanib at D0, D8, D15 and D22. B/ Graph represents evolution of [^68^Ga]Ga-NODAGA-collagelin lung uptake (% ID/g) at all time points. Results are presented as median ± interquartile range, n = 4 for all groups. Black arrow represents the start of treatments. C/ Graph represents evolution of mean lung density (HU) at all time points. Results are presented as median ± interquartile range, n = 4 for all groups. Stars (*) are representative of statistical comparison between time points for each group and hashs (#) are representative of statistical comparison between the groups at each time point. Differences between groups were compared using Kruskal-Wallis non-parametric ANOVA. *^(#)^p < 0.05. Black arrow represents the start of treatments. D/ Graph represents the intensity of picrosirius red staining on lung section from of NaCl- and BLM-receiving mice treated or not with nintedanib at D22. Results are presented as median ± interquartile range, n = 4 for all groups. E-F/ Representative Masson trichrome (E) and PSR (F) stainings of lung sections from NaCl- and BLM-receiving mice treated or not with nintedanib (D22), scale bar = 200µm. A-D/ Results are presented as median ± interquartile range, n = 4 for all groups (B, C and D). Stars (*) are representative of comparison either between time points for each group (B, C) or with NaCl group (D). Hashs (#) are representative of statistical comparison either between groups at each time points (B, C) or with BLM and BLM + nintedanib groups (D). Differences between groups were compared using Kruskal-Wallis non-parametric ANOVA. *^(#)^p < 0.05, **^(##)^p < 0.01.

NaCl and BLM-receiving mice treated or not with nintedanib underwent longitudinal imaging with [^68^Ga]Ga-NODAGA-collagelin successively at D8, D15 and D22 (Figure S3B). Lung uptake of [^68^Ga]Ga-NODAGA-collagelin increased at early stage (D8) in BLM-receiving mice and remained higher compared to controls up to D22 (Figure 3A-B). Interestingly, in BLM-receiving mice nintedanib treatments dramatically decreased [^68^Ga]Ga-NODAGA-collagelin lung uptake at D15 and D22 (Figure 3A-B). Similarly, [^68^Ga]Ga-NODAGA-collagelin lung-to-blood and lung-to-muscle ratio increased at D8 in BLM-receiving mice and remained higher compared to controls up to D22 (Figure S4A). Both ratio were significantly decreased by nintedanib (Figure S4A). In parallel, MLD measured on CT significantly increased from D8 to D22 in BLM-receiving mice and this effect was prevented by nintedanib treatments (Figure 3C). Importantly, collagen quantification on lung sections confirmed that nintedanib reduced collagen content in BLM-receiving mice treated with nintedanib compared with mice receiving vehicle at D22 as well as fibrotic lesions (Figure 3D-E-F).

### *In vivo* [^68^Ga]Ga-NODAGA-collagelin PET imaging is a useful tool to monitor tofacitinib efficacy

Beyond IPF, other progressive fibrosing interstitial lung diseases (ILDs) are often associated with connective tissue diseases and may develop in ∼18-32% of patients with ILDs. As IPF, progressive fibrosing ILDs also display a poor prognosis and a lack of recognize antifibrotic treatment apart from nintedanib or pirfenidone. Tofacitinib (already approved for rheumatoid arthritis, psoriatic arthritis and hemorrhagic rectocolitis) is a drug currently underway with a phase 2 (NCT05246293) and phase 4 (NCT04311567) clinical trials in patients with progressive fibrosing ILDs with anti-fibrotic properties as previously described by our group 16. The efficacy of [^68^Ga]Ga-NODAGA-collagelin to monitor the anti-fibrotic efficacy of tofacitinb was assessed in our preclinical model.

**Figure 4:**
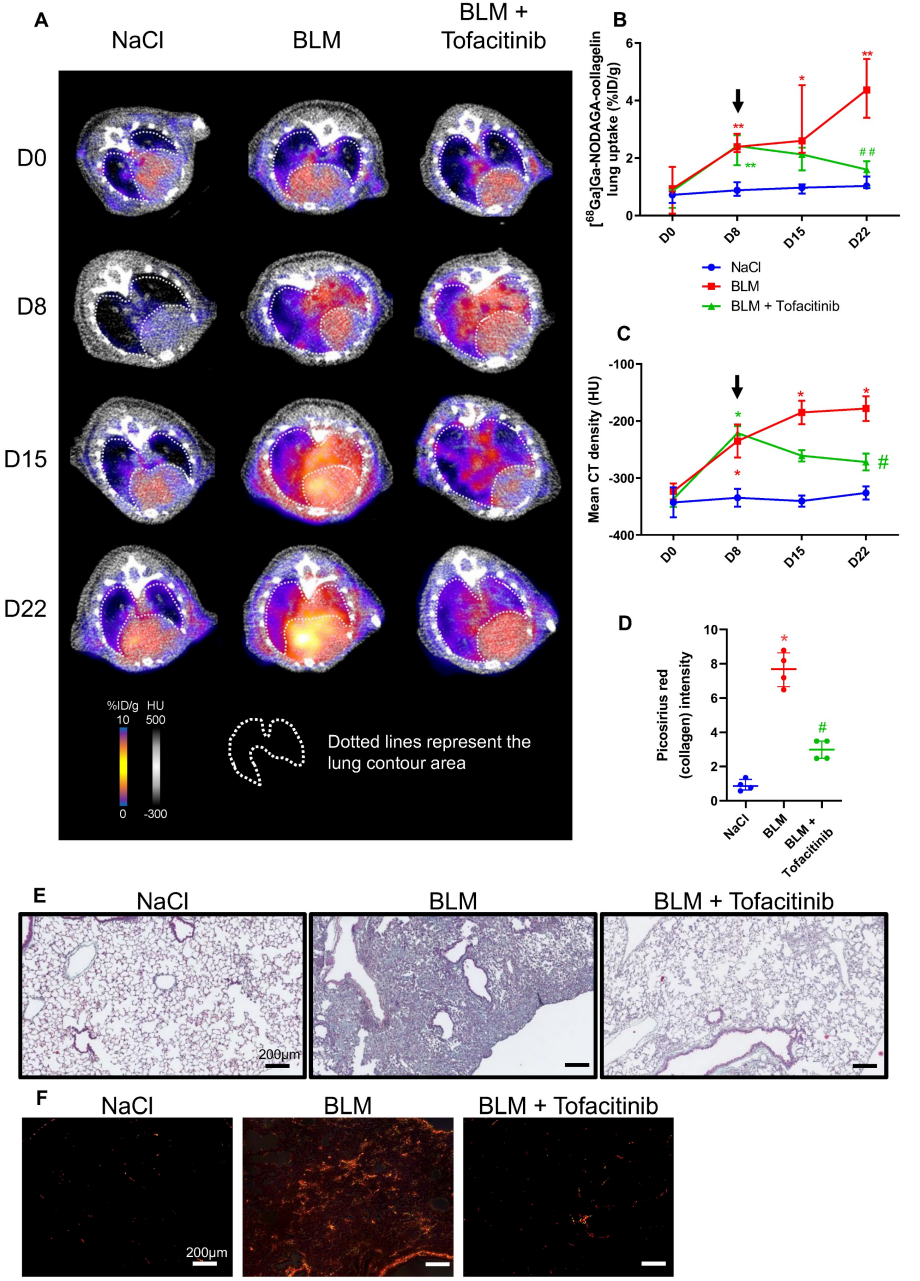
**Tofacitinib reduces lung fibrosis and [^68^Ga]Ga-NODAGA-collagelin lung uptake.** A/ Representative [^68^Ga]Ga-NODAGA-collagelin PET/CT images of NaCl- and BLM-receiving mice treated or not with tofacitinib at D0, D8, D15 and D22. B/ Graph represents evolution of [^68^Ga]Ga-NODAGA-collagelin lung uptake (%ID/g) at all time points. Black arrow represents the start of the treatment. C/ Graph represents evolution of mean lung density (HU) at all time points. Black arrow represents the start of the treatment. D/ Graph represents the intensity of picrosirius red staining on lung section from of NaCl- and BLM-receiving mice treated or not with tofacitinib at D22. E-F/ Representative Masson trichrome (E) and PSR (F) stainings of lung sections from NaCl- and BLM-receiving mice treated or not with tofacitinib (D22), scale bar = 200µm. A-D/ Results are presented as median ± interquartile range, n=4 for all. Stars (*) are representative of comparison either between time points for each group (B, C) or with NaCl group (D). Hashs (#) are representative of statistical comparison either between groups at each time points (B, C) or with BLM and BLM + tofacitinib groups (D). Differences between groups were compared using Kruskal-Wallis non-parametric ANOVA. *^(#)^p < 0.05, **^(##)^p < 0.01.

NaCl and BLM-receiving mice treated or not with tofacitinib underwent longitudinal imaging with [^68^Ga]Ga-NODAGA-collagelin successively at D8, D15 and D22 (Figure S3B). Lung uptake [^68^Ga]Ga-NODAGA-collagelin increased at early stage (D8) in BLM-receiving mice and remained higher compared to control up to D22 (Figure 4A-B).

Interestingly, in BLM-receiving mice tofacitinib treatments dramatically decreased [^68^Ga]Ga-NODAGA-collagelin lung uptake at D22 (Figure 4A-B). Similarly, [^68^Ga]Ga-NODAGA-collagelin lung-to-blood and lung-to-muscle ratio increased at D8 in BLM-receiving mice and remained higher compared to controls up to D22 (Figure S4B). Both ratio were significantly decreased by tofacitinib (Figure S4B). In parallel, MLD measured on CT significantly increased from D8 to D22 in BLM-receiving mice and this effect was prevented by tofacitinib treatments (Figure 4C). Interestingly, collagen quantification on lung sections confirmed that tofacitinib reduced collagen content in BLM-receiving mice treated with tofacitinib compared with mice receiving vehicle at D22 as well as fibrotic lesions (Figure 4D-E-F).

### Early [^68^Ga]Ga-NODAGA-collagelin PET imaging is predictive of fibrosis progression and efficacy of anti-fibrotic therapies

**Figure 5:**
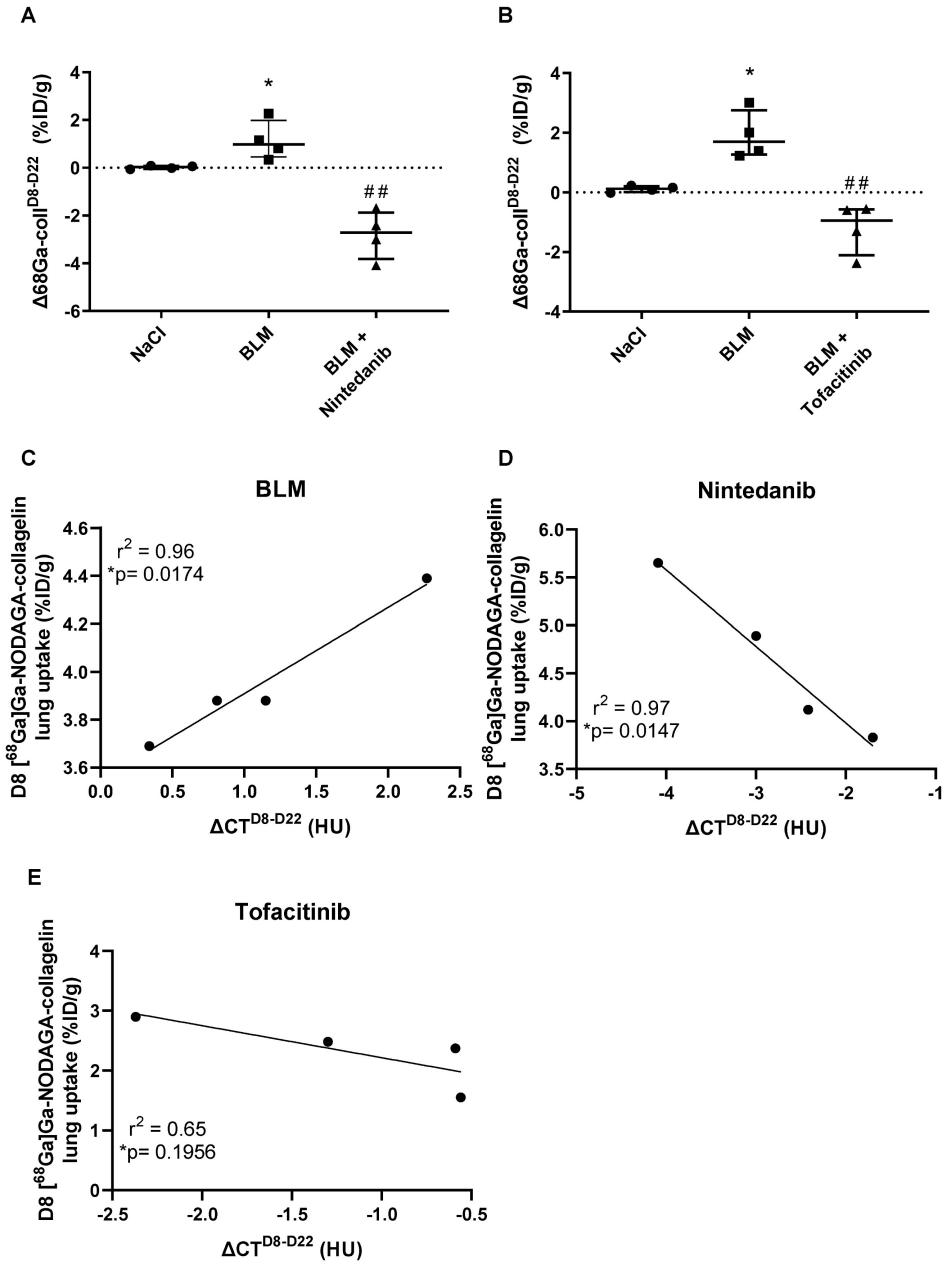
**Predictive value of [^68^Ga]Ga-NODAGA-collagelin PET imaging for lung fibrosis progression and efficacy of nintedanib and tofacitinib.** A/ Variation of [^68^Ga]Ga-NODAGA-collagelin lung uptake between D8 and D22 (Δ68Ga-coll^D8-D22^) in mice receiving NaCl, BLM or BLM + nintedanib. B/ Variation of [^68^Ga]Ga-NODAGA-collagelin lung uptake between D8 and D22 (Δ68Ga-coll^D8-D22^) in mice receiving NaCl, BLM or BLM + tofacitinib. C/ Correlation between variation of mean lung density between D8 and D22 (ΔCT^D8-D22^) and early [^68^Ga]Ga-NODAGA-collagelin lung uptake (D8) in BLM receiving mice. D/ Correlation between variation of mean lung density between D8 and D22 (ΔCT^D8-D22^) and early [^68^Ga]Ga-NODAGA-collagelin lung uptake (D8) in BLM receiving mice treated with nintedanib. E/ Correlation between variation of mean lung density between D8 and D22 (ΔCT^D8-D22^) and early [^68^Ga]Ga-NODAGA-collagelin lung uptake (D8) in BLM receiving mice treated with tofacitinib. A-C/ Results are presented as median ± interquartile range, n = 4 for all groups. Stars (*) are representative of comparison of each group with NaCl group and hashs (#) are representative of statistical comparison of BLM and BLM + ninedanib groups. Differences between groups were compared using Kruskal-Wallis non-parametric ANOVA. Differences between groups were compared using Kruskal-Wallis non-parametric ANOVA. *^(#)^p < 0.05, **^(##)^p < 0.01.

Fibrosis progression as well as efficacy of nintedanib and tofacitinib were quantified using the variation of [^68^Ga]Ga-NODAGA-collagelin lung uptake between D8 and D22 (Δ^68^Ga-coll^D8-D22^). BLM induced an increase in Δ^68^Ga-coll^D8-D22^ demonstrating fibrosis progression between D8 and D22 (Figure 5A-B). Nintedanib and tofacitinib significantly decreased Δ^68^Ga-coll^D8-D22^ in BLM-treated mice showing the anti-fibrotic potential of these drugs (Figure 5A-B). In order to determine the predictive value of early collagen imaging, correlation studies between the variation of mean lung density (ΔCT^D8-D22^) between D8 and D22 and values of [^68^Ga]Ga-NODAGA-collagelin lung uptake at D8 were performed. Interestingly, in BLM-receiving mice, [^68^Ga]Ga-NODAGA-collagelin lung uptake at D8 positively correlated with ΔCT^D8-D22^ showing that lungs with higher [^68^Ga]Ga-NODAGA-collagelin uptake at D8 were those with higher fibrosis progression (Figure 5C). Interestingly, [^68^Ga]Ga-NODAGA-collagelin uptake at D8 negatively correlated with ΔCT^D8-D22^ in nintedanib- and tofacitinib-treated mice demonstrating that lungs with higher [^68^Ga]Ga-NODAGA-collagelin uptake at D8 were those in which nintedanib and tofacitinib showed the best efficacy (Figure 5D-E).

## Discussion

For the diagnosis and follow-up of various diseases, *in vivo* molecular imaging has lately emerged as a key tool in preclinical research, clinical trials and clinical practice. Recently, it was shown by our team and others that longitudinal CT-scan was a viable technique to track the severity and evolution of lung fibrotic regions in preclinical BLM-induced lung fibrosis 17-20. By demonstrating an elevated MLD during BLM-induced fibrosis, we confirm these findings here. However, CT-imaging has been shown to be ineffective as a predictive tool for disease progression and more specific imaging biomarkers need to be investigated 20. Lung fibrosis is characterized by an excessive and abnormal collagen deposition. As a result, *ex vivo* lung tissue collagen quantification is frequently employed to assess the degree of fibrosis in animal models. In order to detect collagen *in vivo*, we used [^68^Ga]Ga-NODAGA-collagelin as a probe for PET imaging that mainly targets collagen I and III 10, 11. Our results demonstrating an increase in [^68^Ga]Ga-NODAGA-collagelin lung uptake from D8 to D22 in BLM-receiving mice are in accordance with other collagen-binding probes such CBP8 7, EP3533 21, CNA35 22 or collagen hybridizing peptides 23 which demonstrate a gradual increase in organ uptake in correlation with fibrosis severity. While [^68^Ga]Ga-NODAGA-collagelin can bind to collagen I, III and laminin 10, CBP8 and EP3533 are collagen I-specific probes, collagen hybridizing peptides only recognize denatured collagens and CNA35, which also binds to collagen I, III and IV, is a fluorescent probe with no nuclear imaging applications. For instance, Désogère *et al*. showed similar results with a collagen I-specific PET probe, CBP8 also radiolabelled with gallium-68 ([^68^Ga]Ga-NODAGA-CBP8), with high specificity of [^68^Ga]Ga-NODAGA-CBP8 for pulmonary fibrosis *in vivo* in BLM-treated mice 7. In addition, our findings with [^68^Ga]Ga-NODAGA-collagelin were in accordance with [^68^Ga]Ga-NODAGA-CBP8 lung uptake, which correlated with lung collagen content and showed sensitivity to monitor response to treatment in BLM-induced lung fibrosis 7. Very recently, [^68^Ga]Ga-NODAGA-CBP8 has been shown to display favourable in-human characteristics and dosimetry suggesting that it could therefore be used for non-invasive collagen imaging across a range of human fibrotic diseases 6, 9. The first-in-human study with [^68^Ga]Ga-NODAGA-CBP8 demonstrated encouraging results with an increase in lung uptake in IPF patients found in fibrotic lung regions determined by CT and also in regions where the lung appeared to be normal on CT 9. These findings, that need to be further confirmed in larger studies, reinforce the concept of using collagen PET tracers to assess early lung fibrosis in patients. Of note, recent data may suggest that the use of [^68^Ga]Ga-NODAGA-collagelin which targets both collagen I and III may be of great interest specifically in IPF in which a specific collagen III upregulation has been shown 24.

The increase in collagen III by myofibroblasts in IPF has been demonstrated to be correlated to disease progression, and as a potential serum biomarker for progressive disease phenotypes in IPF patients 25-27. In addition, the turnover of collagen III has been shown to be modulated by nintedanib reinforcing the potential of collagen-III targeting probes to monitor nintedanib efficacy in patients with IPF 28. In addition to collagen I and III, [^68^Ga]Ga-NODAGA-collagelin has been shown to bind to laminin 10, another ECM component that has been linked to lung fibrosis development 29. Further clinical evaluations are needed to determine whether imaging probes targeting collagen I and III, as well as other ECM components, show similar or better clinical results that collagen-I specific probes.

Our results uncover a rather heterogeneous circulating amount of [^68^Ga]Ga-NODAGA-collagelin which tends to increase in fibrotic animals receiving BLM. While this observation may require deeper analysis, we can hypothesize that [^68^Ga]Ga-NODAGA-collagelin is able to bind to circulating collagen degradation fragments that have been demonstrated to be increased during lung fibrosis 30, 31. This finding may have an impact on the clinical application of such imaging probes as it may induce an increase in radioactive dose in off-target organs such as the heart.

The main clinical challenge in progressive fibrosing ILDs including IPF is the unpredictable evolution of lung fibrosis and the impossible monitoring of anti-fibrotic therapy efficacy. Our results demonstrate that [^68^Ga]Ga-NODAGA-collagelin allows the accurate monitoring of collagen decrease following nintedanib and tofacitinib in accordance with CT imaging and histology. Further, our findings demonstrate, for the first time, that collagen imaging with [^68^Ga]Ga-NODAGA-collagelin may be a relevant tool to predict disease progression and efficacy of anti-fibrotic therapy. Longitudinal imaging allowed us to highlight that early [^68^Ga]Ga-NODAGA-collagelin lung uptake (at D8) strongly correlated with lung fibrosis progression from D8 to D22 in BLM-receiving mice. Animals with higher early [^68^Ga]Ga-NODAGA-collagelin lung uptake corresponded to animals with the most severe lung fibrosis progression. In addition, early [^68^Ga]Ga-NODAGA-collagelin lung uptake was also correlated with the efficacy of approved (nintedanib) and anti-fibrotic drugs under investigation for lung fibrosis (tofacitinib) 16. Tofacitinib is a known JAK inhibitor that has been shown to have anti-fibrotic properties in animal models 16 and which is under investigation in the ongoing clinical trials PULMORA (NCT04311567) and RAILDTo (NCT05246293), which aim at demonstrating the efficacy of tofacitinib to reduce lung fibrosis and improve pulmonary function in non-IPF progressive pulmonary fibrosis. The small sample size in our *in vivo* experiments may be a limitation of our study and warrant further *in vivo* confirmation to fully determine the predictive value of early collagen imaging for disease progression and evaluation of therapy efficacy. Nevertheless, these original findings may be of crucial importance for the personalized management of patients with progressive fibrosing ILDs including IPF and to help therapeutic decision to ultimately improve the outcome of patients.

While our preclinical results are certainly promising, their relevance for human progressive lung fibrosis needs further investigation. Indeed, BLM-induced fibrosis may show some important limitations for clinical translation regarding the accumulation of extracellular matrix components. For instance, in our study BLM induces a massive deposition of collagen in the lungs which may be sparser and more heterogeneous in IPF patients at various stage of the disease. In addition, the number of animals used in our study remains modest and further confirmation with larger cohort may be required. Nevertheless, our data on human lung slices highlight the translational potential of [^68^Ga]Ga-NODAGA-collagelin. Our findings demonstrate that the accumulation of a collagen-targeted probe is upregulated in lung fibrosis and can predict disease progression and therapy efficacy in experimental fibrosis. This constitutes a first step in the validation of this radiotracer for further investigations in humans. For instance, collagen-targeted nuclear imaging scanning could serve as a second-line imaging technique when the gold standard CT-imaging is unclear or difficult to interpret.

## Supplementary Material

Supplementary methods and figures.
