# Supplementary material for: Collagen-targeted PET imaging for progressive experimental lung fibrosis quantification and monitoring of efficacy of anti-fibrotic therapies
Source: Theranostics. 2025 Jan 13;15(6):2092–103. doi: 10.7150/thno.106367 (PMC11840721; doi:10.7150/thno.106367)
Supplement: Supplementary file 1 — Supplementary methods and figures. [file thnov15p2092s1.pdf]

Title: Collagen-targeted PET imaging for progressive experimental lung fibrosis quantification and monitoring of efficacy of anti-fibrotic therapies

Authors: Alexandre Magno Maneschy Dias[1], Olivier Burgy[2,3,4], Mathieu Moreau[5], Victor Goncalves[5], Lenny Pommerolle[2,4], Romain Douhard[1], Alan Courteau[1,5], Alex Helbling[1], Mélanie Guillemin[1], John Simonet[1], Alexandra Oudot[1], Carmen Garrido[2,4,7], Philippe Bonniaud[2,3,4,6*], Françoise Goirand[2,3,4], Bertrand Collin[1,4,5], Pierre-Simon Bellaye[1,2,3*].

Affiliations:
[1]Centre Georges-François Leclerc, Service de Médecine Nucléaire, Equipe IMATHERA, 1 rue du professeur Marion, Dijon, France.
[2]INSERM U1231, Equipe HSP-pathies, Faculty of Medicine and Pharmacy, University of Bourgogne-Franche Comté, Dijon, France.
[3]Centre de Référence Constitutif des Maladies Pulmonaires Rares de l'Adultes de Dijon, réseau OrphaLung, Filère RespiFil, Centre Hospitalier Universitaire de Bourgogne, Dijon, France.
[4]Université de Bourgogne, UFR santé, 21000 Dijon France.
[5]Institut de Chimie Moléculaire de l'Université de Bourgogne, UMR CNRS 6302, Université de Bourgogne Franche-Comté, 21000 Dijon, France.
[6] Institut Universitaire du Poumon Dijon-Bourgogne, CHU Dijon, Dijon, France.
[7]Centre Georges-François Leclerc, Early Phase Unit, Dijon, France

*Corresponding authors. Email: philippe.bonniaud@chu-dijon.fr, psbellaye@cgfl.fr

One Sentence Summary: Collagen-targeted PET imaging allows an accurate quantification of experimental lung fibrosis (LF) as well as a promising tool to monitor the efficacy of anti-fibrotic therapies towards personalized medicine in LF.

Supplemental material

Supplemental method

Human tissue samples

Lung tissue samples (n = 4) were obtained by open lung biopsy (FIRH, Hamilton, Canada). Idiopathic pulmonary fibrosis (IPF) was diagnosed according to the American Thoracic Society/European Respiratory Society consensus criteria, including clinical, radiographic, and characteristic histopathologic features. Control non-IPF lung tissue samples were obtained from smokers who underwent thoracic surgery for localized primary lung carcinoma (biopsy away from cancer lesion, n= 4).

Synthesis of NODAGA-collagelin

Fmoc-protected amino acids, Rink amide resin, and coupling reagents were from Iris Biotech. (*R*)-NODAGA-NHS was provided by CheMatech (Dijon, France). All other chemicals were purchased from Sigma-Aldrich, Acros Organics and Alfa Aesar and used without further purification.

Purifications by semi-preparative HPLC were performed on an UltiMate 3000 system from Dionex (Thermo Scientific) equipped with an UV-visible detector. Fractions of interest were analyzed by HPLC-MS, pooled, concentrated under reduced pressure to remove organic solvents and freeze-dried.

High performance liquid chromatography-mass spectrometry (HPLC-MS) analyses were performed on an UltiMate 3000 system from Dionex (Thermo Scientific) equipped with a DAD detector and coupled to a low-resolution mass spectrometry detector MSQ Plus (Thermo Scientific) with an ESI source. Separation was achieved using an RP Kinetex™ column (Phenomenex) (2.6 µm, 100 Å, 50 × 2.1 mm) with HPLC quality solvents: A: H_2O 0.1% FA and B: MeCN 0.1% FA. Analyses were performed with the following gradient program: 5% to 100% of B in 5 min, 100% B for 1.5 min, 100% to 5% B in 0.1 min and 5% B for 1.9 min, at a flow rate of 0.5 mL/min. The purity of title compounds was determined from the integration of HPLC-MS chromatograms at 214 nm.

The number of TFA counterions was quantified by ionic chromatography. Chromatographic analysis was performed using an ion chromatograph Thermo Scientific Dionex ICS 5000 with a conductivity detector CD (Thermo Scientific Dionex) and a conductivity suppressor ASRS-ultra II 4 mm (Thermo Scientific Dionex). TFA was separated using an Ion Pac AS11-HC analytical column (4 µm, 4 × 250 mm, Thermo Scientific Dionex) equipped with a guard column. The elution of TFA was conducted with a 15 min isocratic elution with 90% eluent A (NaOH 30 mM) and 10% eluent B (H_2O) at a flow rate of 1.5 mL/min and a column temperature of 30°C. HRMS spectra were recorded in positive mode on a mass spectrometer LTQ Orbitrap XL (Thermo Scientific) using an ESI source.

Collagelin (Fig. S1A) was synthesized in 0.1 mmol scale on a Liberty Blue™ automated microwave peptide synthesizer (CEM, USA). The synthesis was performed by using predefined Fmoc/*t*Bu chemistry protocols on Rink amide aminomethyl-polystyrene resin (loading: 0.48 mmol/g), with DIC and OxymaPure in DMF as coupling agents, and a solution of 20% piperidine in DMF for Fmoc-deprotection steps. At the end of the elongation, the resin was treated with a cleavage solution formed of TFA/TIS/DODT/H_2O, 92.5/2.5/2.5/2.5 (*v*/*v*) (5 mL) for 2.5 h. The resin was removed by filtration and the filtrate was concentrated under a flow of nitrogen. The crude, reduced peptide was precipitated in diethyl ether and recovered by centrifugation at 4000 rpm for 10 minutes.

The peptide was then dissolved in TFA (10 mL) and DMSO (1 mL) was added. The solution was stirred at room temperature and the oxidation of collagelin was monitored by LC-MS analyses. After 3 h, TFA was evaporated and the residue was purified by semi-preparative HPLC on a BetaBasic-18 column (Thermo Scientific) (5 µm, 150 Å, 150 × 30 mm) at 20 mL/minute, with HPLC grade eluents (A: H_2O 0.1% TFA, B: MeCN 0.1% TFA) and the following gradient program: 2% of B for 15 min and then from 2% to 50% of B in 50 min. The appropriate fractions were lyophilized to afford the target peptide, collagelin, as a white powder (with 4 TFA as counterions, as determined by ionic chromatography).

To a solution of collagelin (15 mg, 6.40 µmol, 1 equiv) in dry DMF (700 µL) were added DIPEA (15.7 µL, 89.6 µmol, 14 equiv) and (*R*)-NODAGA-NHS (7.0 mg, 9.60 µmol, 1.5 equiv). The mixture was stirred at 35°C until completion of the reaction (2 h) and directly purified by semi-preparative HPLC (gradient

program: 5% of B for 10 min and then from 5% to 60% of B in 50 min). NODAGA-collagelin was isolated as white fluffy powder (10.8 mg, with 5 TFA as counterions, as determined by ionic chromatography). Purity (HPLC-MS) > 96%, t_R = 3.05 min. HRMS (ESI) m/z calculated for calculated for $C_{92}H_{143}N_{29}O_{31}S_3$ $[M + 4H]^{4+}$ 562,49895, found 562.49990.

Radiolabelling

Collagelin was synthesized in 0.1 mmol scale on a Liberty Blue™ automated microwave peptide synthesizer (CEM, USA). The synthesis was performed by using predefined Fmoc/tBu chemistry protocols on Rink amide aminomethyl-polystyrene resin (loading: 0.48 mmol/g), with DIC and OxymaPure in DMF as coupling agents, and a solution of 20% piperidine in DMF for Fmoc-deprotection steps. At the end of the elongation, the resin was treated with a cleavage solution formed of TFA/TIS/DODT/H2O, 92.5/2.5/2.5/2.5 (v/v) (5 mL) for 2.5 h. The resin was removed by filtration and the filtrate was concentrated under a flow of nitrogen. The crude, reduced peptide was precipitated in diethyl ether and recovered by centrifugation at 4000 rpm for 10 minutes.

The peptide was then dissolved in TFA (10 mL) and DMSO (1 mL) was added. The solution was stirred at room temperature and the oxidation of collagelin was monitored by LC-MS analyses. After 3 h, TFA was evaporated and the residue was purified by semi-preparative HPLC on a BetaBasic-18 column (Thermo Scientific) (5 µm, 150 Å, 150 × 30 mm) at 20 mL/minute, with HPLC grade eluents (A: H2O 0.1% TFA, B: MeCN 0.1% TFA) and the following gradient program: 2% of B for 15 min and then from 2% to 50% of B in 50 min. The appropriate fractions were lyophilized to afford the target peptide, collagelin, as a white powder (with 4 TFA as counterions, as determined by ionic chromatography).

To a solution of collagelin (15 mg, 6.40 µmol, 1 equiv) in dry DMF (700 µL) were added DIPEA (15.7 µL, 89.6 µmol, 14 equiv) and (R)-NODAGA-NHS (7.0 mg, 9.60 µmol, 1.5 equiv). The

mixture was stirred at 35°C until completion of the reaction (2 h) and directly purified by semi-preparative HPLC (gradient program: 5% of B for 10 min and then from 5% to 60% of B in 50 min). NODAGA-collagelin was isolated as white fluffy powder (10.8 mg, with 5 TFA as counterions, as determined by ionic chromatography). Purity (HPLC-MS) > 96%, tR = 3.05 min. HRMS (ESI) m/z calculated for calculated for C92H143N29O31S3 [M + 4H]4+ 562,49895, found 562.49990. For ^{68}Ga-collagelin, a gallium generator was eluted and the activity was recovered in a recovery vial. In a LoBind microtube 23mM of radiolabeling buffer (AcONa 1M), 1 mg.mL^{-1} of collagelin (2817 g.mol^{-1}), 5.4 MBq.nmol^{-1} were added. Then, ethanol was added to a corresponding volume of 1/10th of the radiolabeling solution. The pH of the radiolabeling solution had to be between 3.5 and 4. The microtube was placed under agitation at 1000 rpm for 5 min at 80°C. The radiochemical purity was checked by ITLC using a γ radiochromatograph. The stationary chromatography phase was SG (silica gel) where 1µL of solution was deposited. The eluent used was sodium citrate 0.1M pH = 5. In this system, the complexed ^{68}Ga remained at the deposition point while the free ^{68}Ga migrated to the solvent front. Results have been obtained using the software WinScan software. To validate the radiolabeling, the radiochemical purity had to be higher than 90%. The radiolabeling solution was then dissolved with Phosphate-Buffered Saline (PBS) to inject 1.5nmol of collagelin, 5MBq and 100µL per mouse.

The stability of the radioconjugate in plasma was evaluated by incubating 5 MBq in 500 µL of plasma, and performing a radio-HPLC analysis after precipitation of the proteins in acetonitrile. The radiochemical purity of the radioconjugate in plasma was > 88 % after 2 h in plasma.

Supplemental Figures

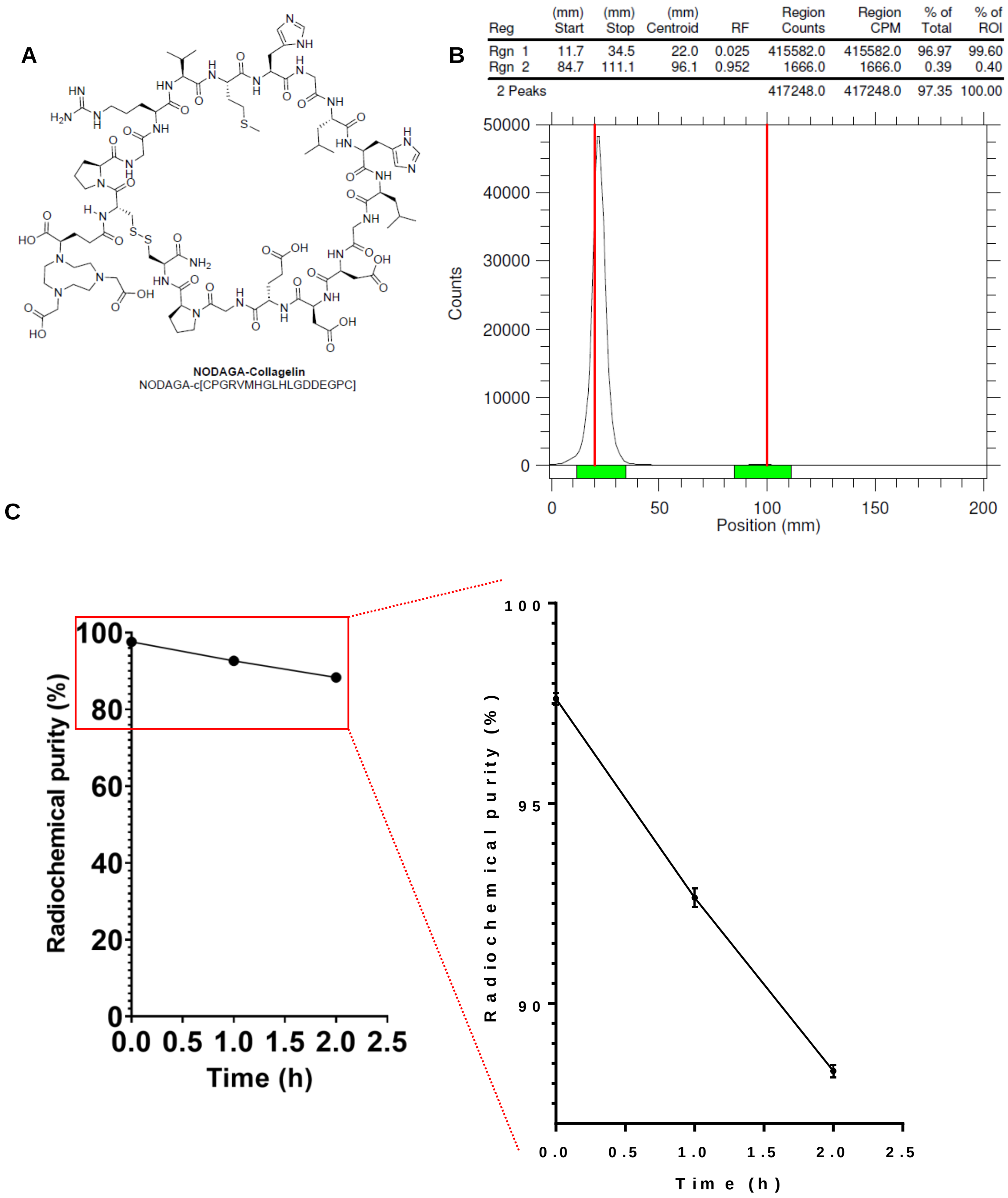

Fig. S1 [^{68}Ga]Ga-NODAGA-collagelin structure, radiolabeling and stability

A/ Chemical structure of *[^{68}Ga]Ga-NODAGA-collagelin. B/ Radiochemical purity* of *[^{68}Ga]Ga-NODAGA-collagelin measured by ITLC. C/ Stability of [^{68}Ga]Ga-NODAGA-collagelin in plasma measured by ITLC.*

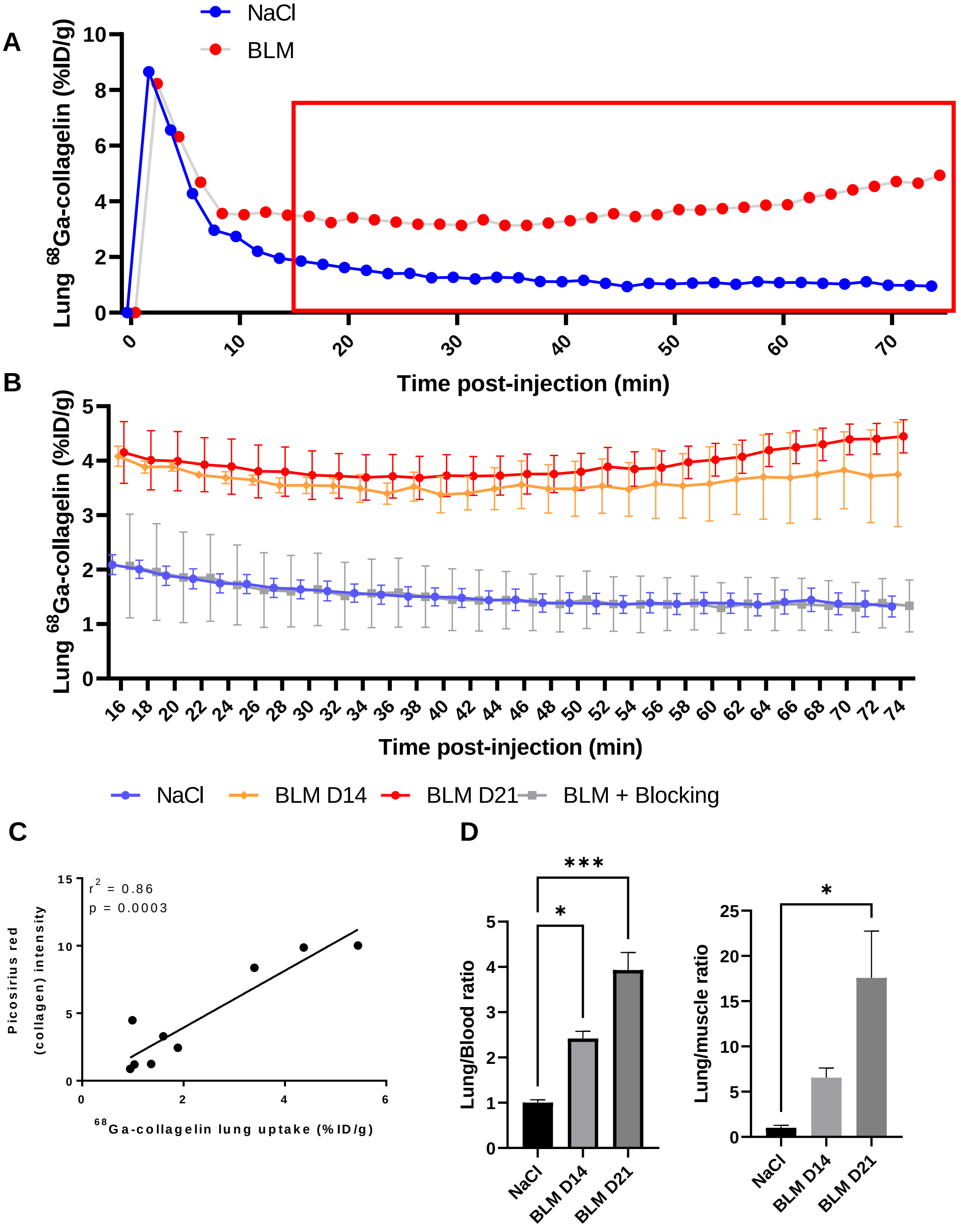

Fig. S2 ^{68}Ga-collagelin is able to detect several stages of BLM-induced lung fibrosis.

A/ Graph represents the dynamic quantification of ^{68}Ga-Collagelin (from 2 min to 75 min p.i.) lung uptake in %ID/g measured by PET of NaCl- (n=1) and BLM-receiving (n=1) mice at D21.

Red frame = optimal window for ^{68}Ga-Collagelin lung uptake quantification. B/ Graph represents the dynamic quantification of ^{68}Ga-Collagelin (from 15 min to 75 min p.i.) lung uptake in %ID/g measured by PET of NaCl- (n=8) and BLM-receiving mice at D14 (n=3), D21 (n=10) and blocking (D21 n=3). C/ Correlation between quantification of ^{68}Ga-Collagelin and Picrosirius red staining of NaCl- (n=1) and BLM-receiving (n=1) mice at D21. D/ Graph represents lung-to-blood and lung-to-muscle ratio measured by gamma counting in NaCl- and BLM-receiving mice at D14 and D21. Results are presented as median ± interquartile range, NaCl, n=15; BLM, D14, n=3; BLM, D21, n=13.

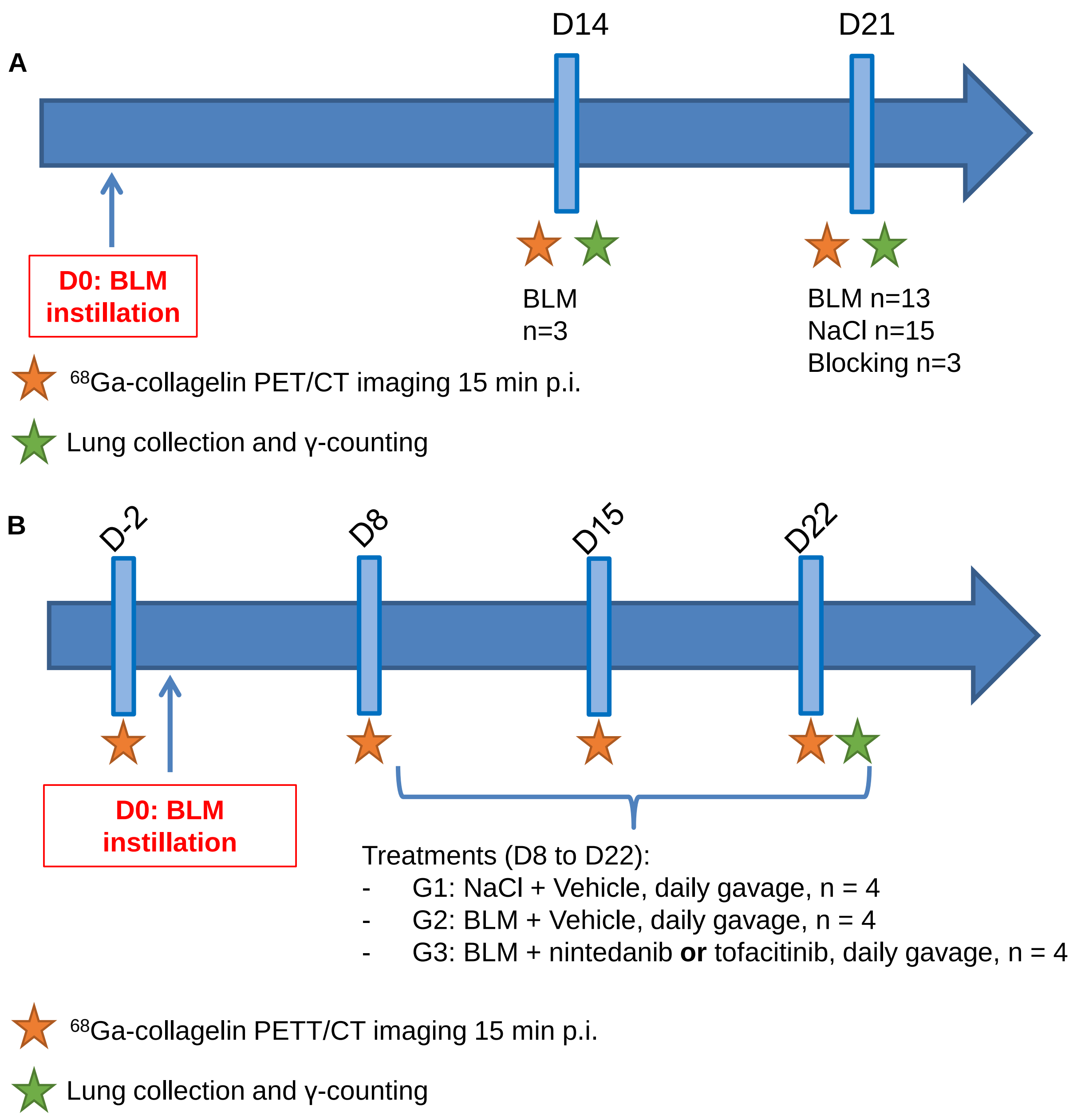

Fig. S3 In vivo experiment protocols

A/ Schematic representation of the design of the study of ^{68}Ga-collagelin imaging in NaCl and BLM-receiving mice at several stage of experimental fibrosis. B/ Schematic representation of the design of the longitudinal study of ^{68}Ga-collagelin imaging with nintedanib or tofacitinib treatments.

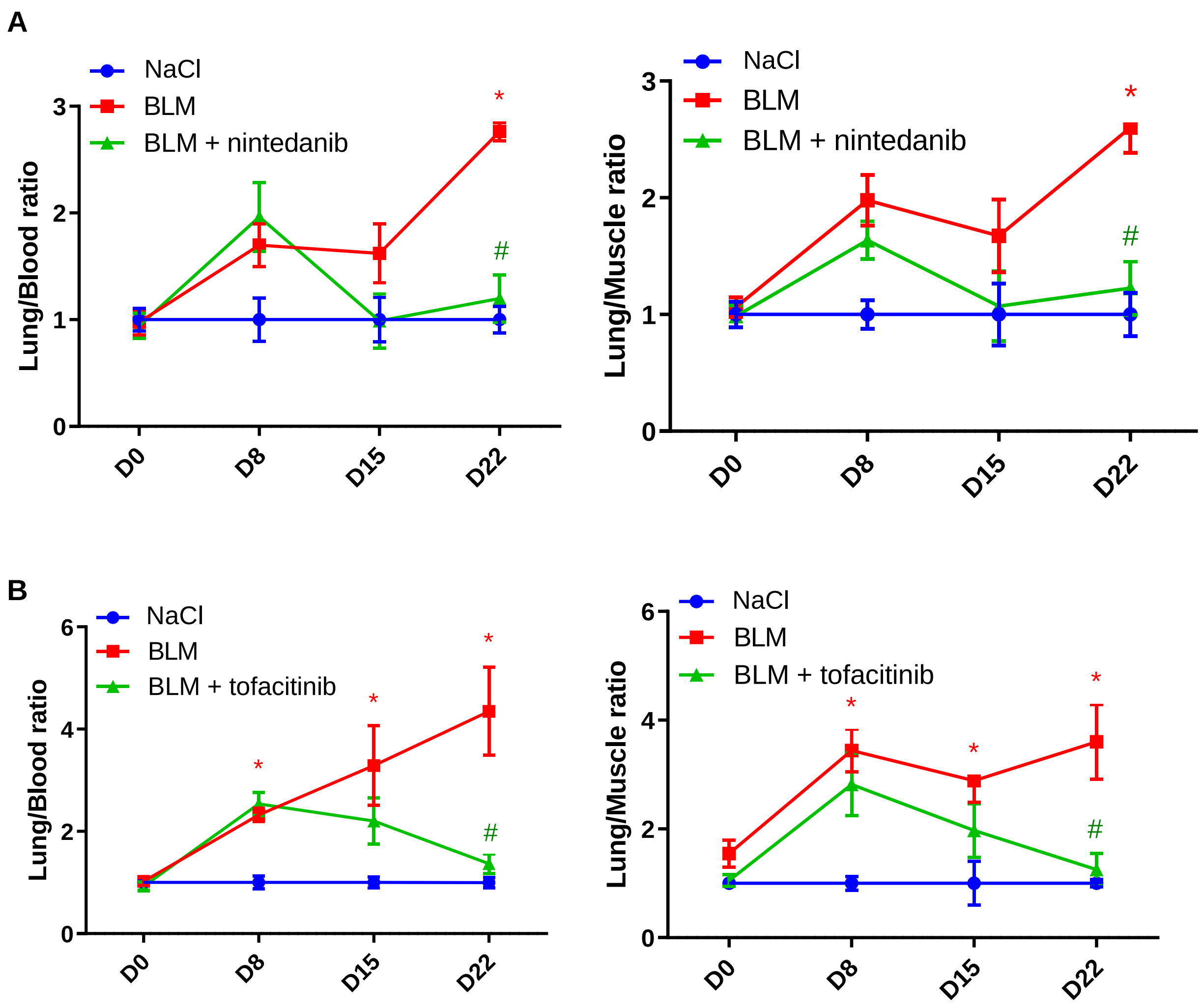

Fig. S4 [^{68}Ga]Ga-NODAGA-collagelin lung-to-blood and lung-to-muscle ratio

A-B/ Graph represents evolution of [^{68}Ga]Ga-NODAGA-collagelin lung-to-blood and lung-to-muscle ratio at all time points in NaCl- and BLM-receiving mice treated or not with nintedanib (A) or tofacitinib (B). Results are presented as median ± interquartile range, n = 4 for all groups. A and B: Stars (*) are representative of comparison between time points for each group. Hashs ([#]) are representative of statistical comparison either between groups at each time points. Differences between groups were compared using Kruskal-Wallis non-parametric ANOVA. *[(#)]$p<0.05$.
